# Hospitals for the Middle Classes

**Published:** 1924-01

**Authors:** Harold Wigg

**Affiliations:** 12, Tanza Road, Hampstead, N.W. 3


### Hospitals for the Middle Classes.

SIR,—There is growing evidence of a tendency to divert the voluntary hospitals of the country from their original purpose. The conversion of a number of beds provided for the poor to pay-beds for those in better circumstances of life is under the consideration of the management boards of many hospitals. In some hospitals pay beds have already been set aside, and for these fixed fees are charged which are within the means of those only who have moderately good incomes.

For years past the hospitals have been invaded by those in quite good circumstances of life, because, being insufficiently well provided to pay for accommodation in expensive nursing homes and the fees of consultants, no other alternative has been possible. It does not follow because the number of necessitous persons in the hospitals is now less than it was, that there are fewer necessitous people. That is obviously not the case. Some of the great hospitals of the country have waiting lists so long that many applicants fail to obtain the treatment required. Where, then, are the necessitous poor accommodated? The truth is that they are driven to seek relief at the workhouse infirmaries, which are intended for the destitute. A great change has taken place in the character of the work carried on by the Poor Law infirmaries in recent years, a change which perhaps in some measure is due to altered circumstances consequent upon the war. In past years these institutions provided mainly for those who were destitute or those whose chronic illnesses had brought them to destitution. To-day may be found in the wards of these infirmaries a large proportion of patients drawn from the industrial population who require hospital treatment for acute illnesses. Many Poor Law infirmaries are now specially equipped to provide this need, and retain the services of highly skilled surgeons and physicians.

Thus it would appear that there is a danger that the work hitherto carried on by the voluntary hospitals will eventually be transferred to the Poor Law infirmaries and municipal hospitals, and that the voluntary hospitals will become pay hospitals for the middle classes. Side by side with this change will appear a change in the relationship between the staffs of the hospitals and the patients. It is obvious that the physicians and surgeons could not continue to give their services free of charge to those who are in a position to pay reasonable fees; and it is equally certain that they would not act as honorary officers to Poor Law or municipal hospitals.

Little need be said in support of the urgent need for establishing middle-class hospitals. While many who can afford to pay moderate fees for their maintenance and treatment are forced to seek relief in the voluntary hospitals, a still larger number are obliged to jeopardise their financial stability in order to meet elsewhere the heavy expenses which serious illness thrusts upon them. For the ordinary needs of life it is possible to shop in the markets which suit the purse, but for the care and attention requisite for serious illnesses no such choice is available for a large section of the community.

Yet suitable hospital accommodation could be provided by the middle classes themselves at a cost involving no individual sacrifice. The medical profession have ever been ready to treat, free of charge, the sick poor, and they would be equally willing to extend the necessary consideration to those in better circumstances. The establishment of suitable hospitals providing accommodation at charges graduated to suit the patients' purses, which should be open to the practice of all members of the medical profession willing to render professional service at fees graduated in a like manner, would seem to be a rational method of solving the problem.

A hospital of this character should provide small wards and single rooms suitably arranged on floors for medical, surgical and maternity cases and for children. It should be completely equipped as a general hospital, with special departments and staffed by resident and departmental medical officers. It should provide a training school for nurses, for the employment of trained nurses only, while not increasing efficiency, would raise unduly the cost of nursing.

The inclusive charges for accommodation might range from £2 2s. in small wards to £10 10s. weekly in the best single rooms. If the charges were so arranged as to produce an average payment of £5 5s. per week per bed, or even less, the hospital would be self-supporting. It should be possible with efficient management to maintain such a hospital without difficulty upon this basis of payment. For instance, the average cost of maintaining a bed at a general hospital is now about £3 3s. a week. Thus there should be ample margin for increased expenditure upon the possible additional requisites of a private hospital and upon the maintenance of the nursing staff, which must of necessity be larger *pro rata* to patients than in an ordinary general hospital.

The expenditure upon buildings would be so considerable as seriously to endanger the success of the scheme if the capital were acquired otherwise than on philanthropic terms, for interest on capital would increase the average maintenance charges payable by the patients very considerably. This could, however, be raised by the collection of small periodical subscriptions from the very large section of the community requiring the hospital accommodation; and hospitals could be erected in selected centres of population as funds permitted.

Provision might be made for allocating a small percentage of the sums subscribed to the maintenance of the existing voluntary hospitals, which in times of emergency are open to all classes. Such a provision would form a link between the two systems and do much toward conserving the spirit which created the voluntary hospitals.

12, Tanza Road, Hampstead, N.W. 3. HAROLD WIGG.

